# MiR-4261 Regulates Granulosa Cell Proliferation Mediated by Progesterone via FZD5 in Human Ovarian Granulosa Cell Line

**DOI:** 10.3390/genes17101297

**Published:** 2026-10-18

**Authors:** Jiao Yu, Yangyang Zhang, Yanrong Kuai, Sheng Wang, Yang Xu

**Affiliations:** Department of Obstetrics and Gynecology, Peking University First Hospital, Beijing 100034, China

**Keywords:** progesterone, follicular microenvironment, miR-4261, FZD5

## Abstract

**Background:** The effect of different controlled ovarian hyperstimulation (COH) protocols on the microenvironment of follicular growth may be reflected by microRNA (miRNA) expression profiles. Downregulation of miR-4261 when progesterone is added in the follicular phase has been reported in our previous research. The purpose of this study is to observe how miR-4261 regulates the function of granulosa cells, so as to find the specific pathway and mechanism of progesterone acting on the follicular microenvironment. **Results:** The human ovarian granulosa cell line (KGN cells) is used to conduct cell experiments as granulosa cells. Compared with microRNA-negative control (miR-NC), KGN cells transfected with the miR-4261 mimic showed progressively reduced proliferation at 24, 48, and 72 h (*p* < 0.01). The levels of Frizzled 5 (FZD5) mRNA and protein were also significantly reduced (*p* < 0.01). In the dual-luciferase reporter gene experiment, the fluorescence value of KGN cells co-transfected with wild type-FZD5 (wt-FZD5) and the miR-4261 mimic/inhibitor decreased/increased (*p* < 0.01), and there was no significant difference in fluorescence value between mutant-FZD5 (mut-FZD5) and the miR-4261 mimic/inhibitor co-transfected KGN cells (*p* > 0.05). The proliferation activity of KGN cells transfected with small interfering FZD5 (siFZD5) decreased significantly at 48 h (*p* < 0.01) compared with the control. Compared with KGN cells co-transfected with siFZD5 and miR-NC, there was no significant difference in the proliferation activity of KGN cells co-transfected with siFZD5 and miR-4261 inhibitor (*p* > 0.05). **Conclusions:** The down-regulation of miR-4261 can promote the proliferation of granulosa cells. The knockdown of FZD5 can inhibit the proliferation of granulosa cells. FZD5 is the direct downstream target gene of miR-4261 in KGN cells. miR-4261 can regulate granulosa cell proliferation by targeting FZD5.

## 1. Background

Controlled ovarian hyperstimulation (COH) constitutes a core component of in vitro fertilization (IVF). COH aims to induce synchronous growth, development, and maturation of multiple follicles within a pharmacologically controllable window to retrieve a greater number of high-quality oocytes. Apart from the luteal-phase stimulation protocol and progestin-primed ovarian stimulation (PPOS) protocol, progesterone is not administered during ovarian stimulation in other COH regimens. Follicles and their microenvironment are regulated solely by gonadotropins and estrogens under these conventional protocols. By contrast, both luteal-phase stimulation and PPOS rely on the combined actions of progesterone and estrogen. The progesterone applied in luteal-phase stimulation originates from the post-ovulatory corpus luteum, whereas progesterone used in the PPOS protocol is exogenously supplemented. Progesterone administered during COH in the PPOS protocol suppresses the luteinizing hormone surge and protects the endometrium for women with endometrial carcinoma/atypical endometrial hyperplasia (EC/AEH) who receive fertility treatment [1]. A meta-analysis found that the duration of stimulation, gonadotropin consumption, oocyte yield, live birth rate, ongoing and clinical pregnancy rate per embryo transfer cycle were similar with progesterone and gonadotropin-releasing hormone (GnRH) analogues in COH process [2]. A newly published retrospective clinical study showed that the PPOS protocol could increase the rate of excellent embryos without increasing the recurrence rate of early EC/AEH [3], suggesting that the PPOS protocol is a feasible strategy for fertility treatment of EC/AEH patients.

Granulosa cells (GCs) surround the oocyte and play a key role in supporting follicle growth and oocyte development. The proliferation-apoptosis balance of GCs plays an extremely important role in the follicular microenvironment, oocyte maturation, and embryo formation. MicroRNAs (miRNAs) can target important ovarian function regulators, such as progesterone receptors, aromatase, and follicle-stimulating hormone receptors, which play an important role in transmitting information between oocytes and surrounding supporting cells. At present, there have been many published reports on the regulation of miRNAs and polyendocrine metabolic ovarian syndrome (PMOS) [4], premature ovarian failure (POF) [5] and other diseases, and the expression changes of follicular fluid miRNAs have a related predictive effect on embryo quality [6]. There is no relevant report on the effect of the specific COH protocol on miRNA expression in GCs. There is sufficient theoretical support to further explore the effects of disease states or ovulation induction drugs on the follicular microenvironment through the expression changes of miRNA in GCs.

Progesterone levels in the follicular phase are low, and normal follicle growth and the follicular microenvironment under the action of the conventional COH protocol are not affected by progesterone. How the PPOS protocol adding progesterone during the follicular phase affects the follicular microenvironment and its specific mechanism is the basis of our research. The effect of progesterone supplementation on GCs miRNA expression profiles in the follicular phase has been explored by using GCs miRNA sequencing and PCR validation with the expanded sample size. Downregulation of miR-4261 and upregulation of miR-6869-5p have been reported in our previous research [7]. However, the functional roles of miR-4261 and miR-6869-5p in regulating ovarian granulosa cell function, folliculogenesis, and steroidogenesis remain uncharacterized. In the previous experimental results of miRNA sequencing, miR-4261 had a higher fold difference than miR-6869-5p between the PPOS group and the control group. The miR-4261 with a higher differential multiple was used for the next cell function experiment to understand its regulation on the function of granulosa cells, so as to find the specific pathway and mechanism of progesterone acting on the follicular microenvironment.

## 2. Material and Methods

### 2.1. Study Design, Population, and Ethical Considerations

This study is a prospective study, and the research object is human ovarian granulosa cell line (KGN cells), purchased from Xiamen IMMOCELL Biotechnology Co., Ltd. (Xiamen, China) and routinely passaged.

KGN cells model luteinization-competent human granulosa cells of the preovulatory follicle, as evidenced by their expression of the Follicle-Stimulating Hormone Receptor/Luteinizing Hormone/Choriogonadotropin Receptor (FSHR/LHCGR), robust progesterone synthesis in response to gonadotropins, and transcriptomic similarity to primary granulosa cells collected at the time of hCG administration in IVF cycles, which can effectively simulate the biological characteristics of human primary ovarian granulosa cells.

### 2.2. Cell Culture and Transfection

In this study, KGN cells were cultured in complete medium consisting of DMEM/F12 medium supplemented with 10% fetal bovine serum. The cells were maintained in a constant-temperature incubator with 37 °C and 5% CO humidified atmosphere to ensure normal growth and proliferation. To investigate the biological function of miR-4261, we set up three experimental groups in this study: the miR-4261 mimic overexpression group, miR-4261 inhibitor knockdown group, and miR-negative control (miR-NC) group. The miR-4261 mimic, miR-4261 inhibitor, and corresponding negative control oligonucleotides were all purchased from Beijing Biomed Company (Beijing, China). Cell transfection was performed using Lipofectamine 3000 transfection reagent (Invitrogen, Carlsbad, CA, USA) in strict accordance with the experimental operation specifications formulated in our laboratory. After 48 h of continuous transfection, stable overexpression and knockdown of miR-4261 in KGN cells were successfully constructed, and the transfected cells were collected for subsequent cell function verification experiments. The sequences of the miR-4261 mimic and miR-4261 inhibitor are as follows:
MiR-4261 mimicAGGAAACAGGGACCCAMiR-4261 inhibitorTGGGTCCCTGTTTCCT

The transfected KGN cells were subjected to subsequent cell function experiments.

### 2.3. Cholecystokinin (CCK8) Assay

Transfected KGN cells from the miR-4261 mimic group, miR-4261 inhibitor group, and miR-NC group were inoculated into 96-well culture plates, with six duplicate wells set for each group to ensure experimental repeatability. After routine adherent culture, cell proliferation activity was detected at four time points of 0 h, 24 h, 48 h, and 72 h after transfection. At each preset time point, 10 μL CCK-8 reagent was added to each well for incubation. After 3 h of constant-temperature incubation, the absorbance value of each well at 450 nm wavelength was measured by a microplate reader. In this study, the absorbance value of the miR-NC group at each time point was defined as the baseline value (set as 1), and the relative proliferation activity of KGN cells in the overexpression and knockdown groups was calculated accordingly.

### 2.4. 5-Ethynyl-2′-deoxyuridine (EdU) Assay

5 × 10^4^ cells in each well were seeded into six-well plates and transfected with the miR-4261 mimic, miR-4261 inhibitor and miR-control. After incubation of 48 h, the EdU assay was performed using BeyoClick^TM^ EdU-594 Cell Prolifeation Assay Kit (Beyotime Biotech Inc., Shanghai, China) in accordance with the manufacturer’s instructions. Under an optical microscope, we observed and photographed the cells, in which EdU-positive proliferative cells showed red nuclear fluorescence, and all cell nuclei were counterstained blue. The proliferation level of KGN cells in different groups was compared by counting the proportion of positive cells.

### 2.5. Quantitative Real-Time PCR

Total RNA was isolated from KGN cells using Trizol reagent (Solarbio, Beijing, China). Subsequently, Invitrogen’s reverse transcription kit superscript III was used to complete the reverse transcription. 2× Taq PCR MasterMix (TransGen Biotech, Beijing, China) and SYBR GREEN (TransGen Biotech) were used to carry out the qRT-PCR according to the manufacturer’s instructions. Primers were obtained from Tsingke Biotechnology Co., Ltd. (Beijing, China)., and the sequences were as follows:
**Primers****5′-3′**Frizzled5-F (FZD5-F) GCTCATCCCCAGCGTCAAGTCCAFZD5-RCAGGTTCTGGTTGCCCACGTAGC

Relative gene expression values were calculated using the 2^−△△CT^ method, and U6 was used as the internal reference gene for standardized correction.

### 2.6. Western Blot

The cells were lysed in RIPA buffer (Molecular Dynamics Laboratory) and the protein concentrations were measured by Bicinchoninic Acid (BCA) protein concentration assay kit (Molecular Dynamics Laboratory). Equal amount of protein was separated by Sodium Dodecyl Sulfate–Polyacrylamide Gel Electrophoresis (SDS-PAGE) on a 10% gel and electroblotted onto Polyvinylidene Fluoride (PVDF) membrane (Millipore). Immerse PVDF membrane in 100% methanol for 2 min, rinse with water and the commercial electroporation transfection buffer for 2 min in sequence, and put PVDF membrane in electroporation buffer and probed overnight at 4 °C with primary antibodies. β-ACTIN was used as standard control. After washing three times in Tris-Buffered Saline with Tween 20 (TBST) buffer, the membranes were incubated with the horseradish peroxidase-conjugated secondary antibody for 1 h at 37 °C. Detection by chemiluminescence reaction was performed using ChemiDoc MP Chemiluminescent Imaging System (BIO-Rad, Hercules, CA, USA). Band densities were measured using ImageJ software (Ver 1.5.3) and analyzed using GraphPad Prism software 9.0 (Inc., San Diego, CA, USA).

### 2.7. Co-Transfection

The 3′-untranslated region (3′-UTR) of the human *FZD5* gene, encompassing the predicted miR-4261 binding site (5′-UGCAUU-3′, located at nucleotides 128–133 downstream of the stop codon), was amplified from human genomic DNA and cloned into the *Firefly* luciferase reporter vector pmirGLO (Promega Corporation, Madison, WI, USA), yielding the wild-type construct wt-FZD5-3′-UTR. A site-directed mutant construct (mut-FZD5-3′-UTR) was generated by substituting the seed-matching sequence (5′-UGCAUU-3′ → 5′-ACGTTA-3′) via overlap extension PCR and verified by Sanger sequencing (Sangon Biotech, Shanghai, China).

KGN cells (ATCC^®^ CRL-2927™) were seeded in 96-well plates (1.5 × 10^4^ cells/well) and cultured overnight to achieve ~70% confluence. Cells were co-transfected using Lipofectamine™ 3000 Reagent (Thermo Fisher Scientific, Waltham, MA, USA) with (i) 100 ng of either wt-FZD5-3′-UTR or mut-FZD5-3′-UTR plasmid; (ii) 50 ng of the *Renilla* luciferase control vector pRL-TK (Promega, Madison, WI, USA); and (iii) 50 nM of synthetic RNA oligonucleotides, namely, the miR-4261 mimic, miR-4261 inhibitor, or negative control (miR-NC) (RiboBio Co., Ltd., Guangzhou, China). Six experimental groups were established (*n* = 6 biological replicates per group):

**miR-4261 Mimic****miR-4261-Inhibitor****miR-NC****wt-FZD5****mut-FZD5**A√

√
B
√
√
C

√√
D√


√E
√

√F

√
√

### 2.8. Luciferase

Forty-eight hours post-transfection, cells were lysed, and luminescence was measured sequentially using the Dual-Luciferase^®^ Reporter Assay System (Promega) on a GloMax^®^ Discover microplate reader (Promega). Firefly luciferase activity was normalized to Renilla luciferase activity for each well. Data are presented as mean ± SD of relative luciferase activity, with the wt-FZD5-3′-UTR + miR-NC group set to 100%. All experiments were performed in triplicate and independently repeated three times.

### 2.9. Statistical Analysis

The statistical analyses were performed using GraphPad Prism software version 7.0. The two-tailed un-paired Student’s *t*-test was used to calculate the statistical significance of differences when the samples in two groups met the parameter conditions. One-way and two-way analysis of variance (ANOVA) followed by Least Significant Difference (LSD) were used to assess differences in values for the rest of data. All results were expressed as mean ± standard deviation (SD). A difference of *p* < 0.05 was defined as statistically significant in this study.

## 3. Results

### 3.1. miR-4261 Can Affect Cell Proliferation of KGN Cell

**Figure 1:**
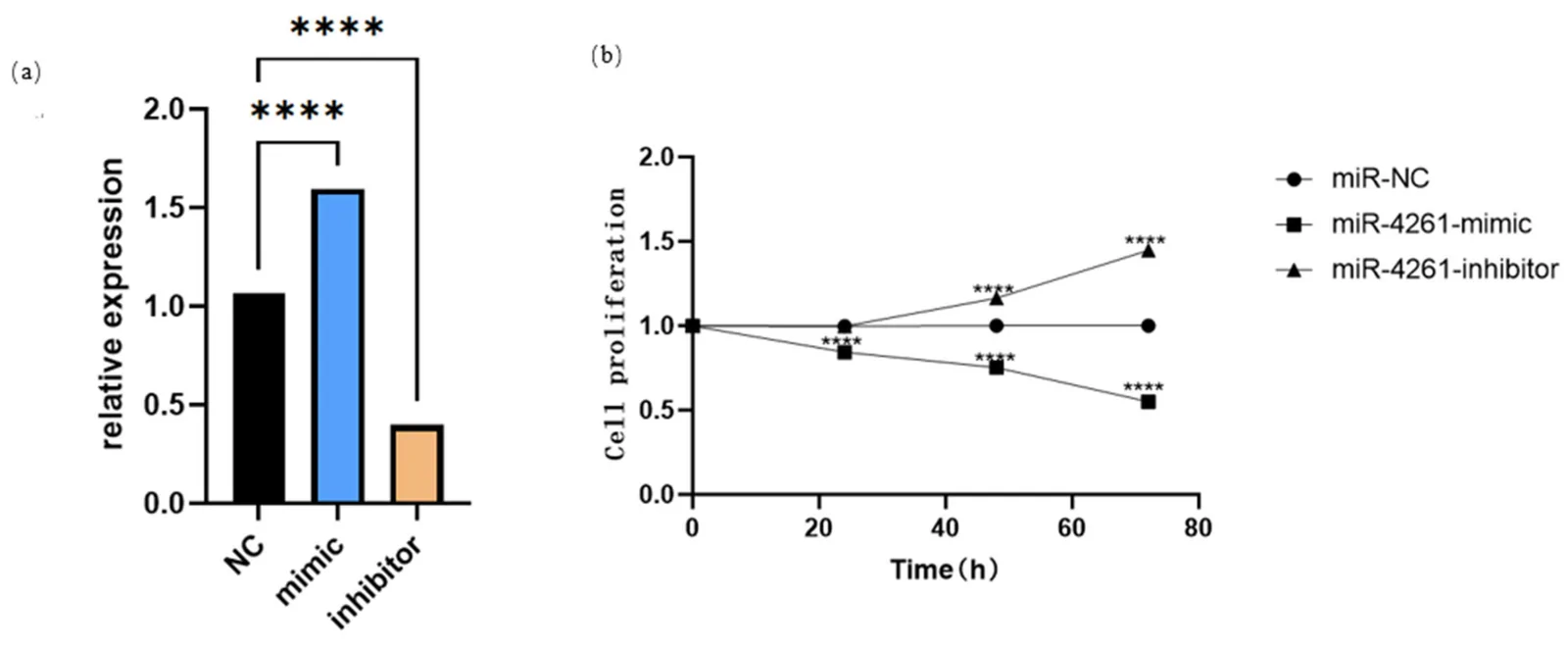
Impact of miR-4261 on KGN cell proliferation. (**a**) qRT-PCR experiment of miR-4261 mimic/inhibitor/NC transfected KGN cells which tested the efficiency of transfection (**** *p* < 0.0001). (**b**) CCK8 assay of miR-4261 expression on KGN cells (**** *p* < 0.0001).

KGN cells were divided into three groups: miR-4261 mimic, miR-4261 inhibitor and miR-NC. Firstly, the transfection efficiency of miRNA was tested by qRT-PCR experiment. qRT-PCR results confirmed that miR-4261 expression was markedly elevated in KGN cells transfected with the miR-4261 mimic, whereas miR-4261 levels were significantly reduced in cells receiving the miR-4261 inhibitor relative to the control group (Figure 1a, *p* < 0.0001). The CCK-8 assay was then used to assess the influence of miR-4261 overexpression on KGN cell proliferation. As illustrated in Figure 1b, no intergroup differences in cellular viability were observed at the 0 h time point. At 24 h, cell viability was significantly lower only in the miR-4261 mimic group compared with the miR-NC group (*p* < 0.0001). At 48 h and 72 h, KGN cells transfected with the miR-4261 mimic exhibited progressively attenuated proliferation relative to miR-NC cells. In contrast, cells transfected with the miR-4261 inhibitor displayed a gradual increase in proliferative capacity, with statistically significant differences detected at these two time points (*p* < 0.0001).

**Figure 2:**
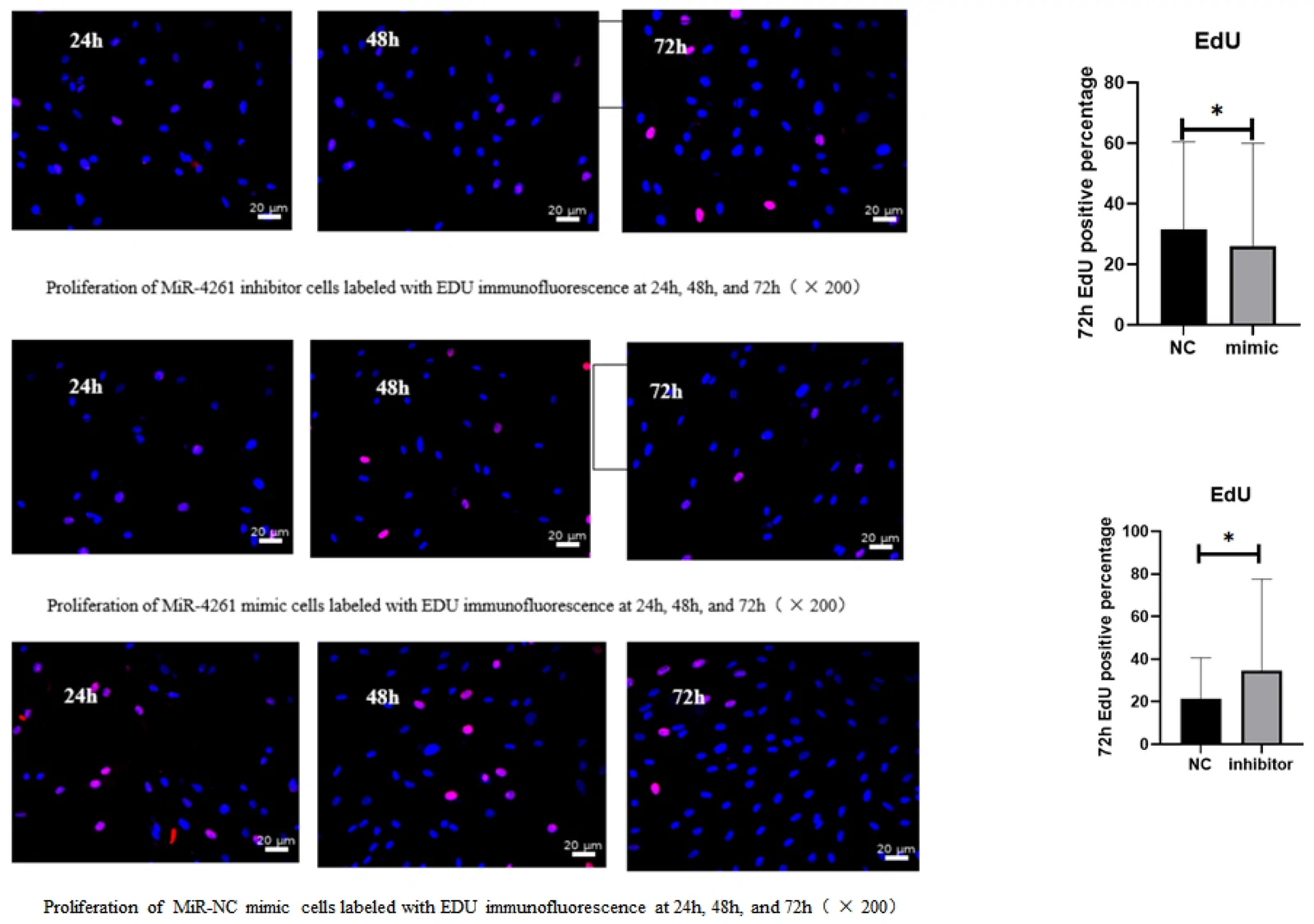
EdU staining of KGN cells transfected with miR-4261 mimic/inhibitor/NC. Data are representative of three independent experiments; five random fields (×200) were captured per condition, with ≥200 nuclei counted per field, and the results were averaged. At 72 h, the percentage of EdU-positive cells in KGN cells transfected with miR-4261 mimic was significantly lower than that in KGN cells transfected with miR-NC (nuclei were stained with DAPI and showed blue, and proliferating cells were stained red with EdU). At 72 h, the percentage of EdU-positive cells in KGN cells transfected with miR-4261 inhibitor was significantly higher than that in KGN cells transfected with miR-NC (* *p* < 0.05).

To further validate the regulatory effect of miR-4261 on KGN cell proliferation, EdU staining was performed to assess cellular proliferative activity. As presented in Figure 2, at 72 h post-transfection, the proportion of EdU-positive proliferating cells was significantly decreased in the miR-4261 mimic group compared with the miR-NC group. Conversely, KGN cells transfected with the miR-4261 inhibitor exhibited a markedly higher percentage of EdU-positive cells relative to the control group. In this assay, cell nuclei were stained blue with DAPI, and proliferating cells were labeled red by EdU staining. Collectively, these results confirm that miR-4261 overexpression suppresses granulosa cell proliferation, whereas miR-4261 downregulation promotes granulosa cell proliferation.

### 3.2. FZD5 Is a Downstream Target Gene of miR-4261

**Figure 3:**
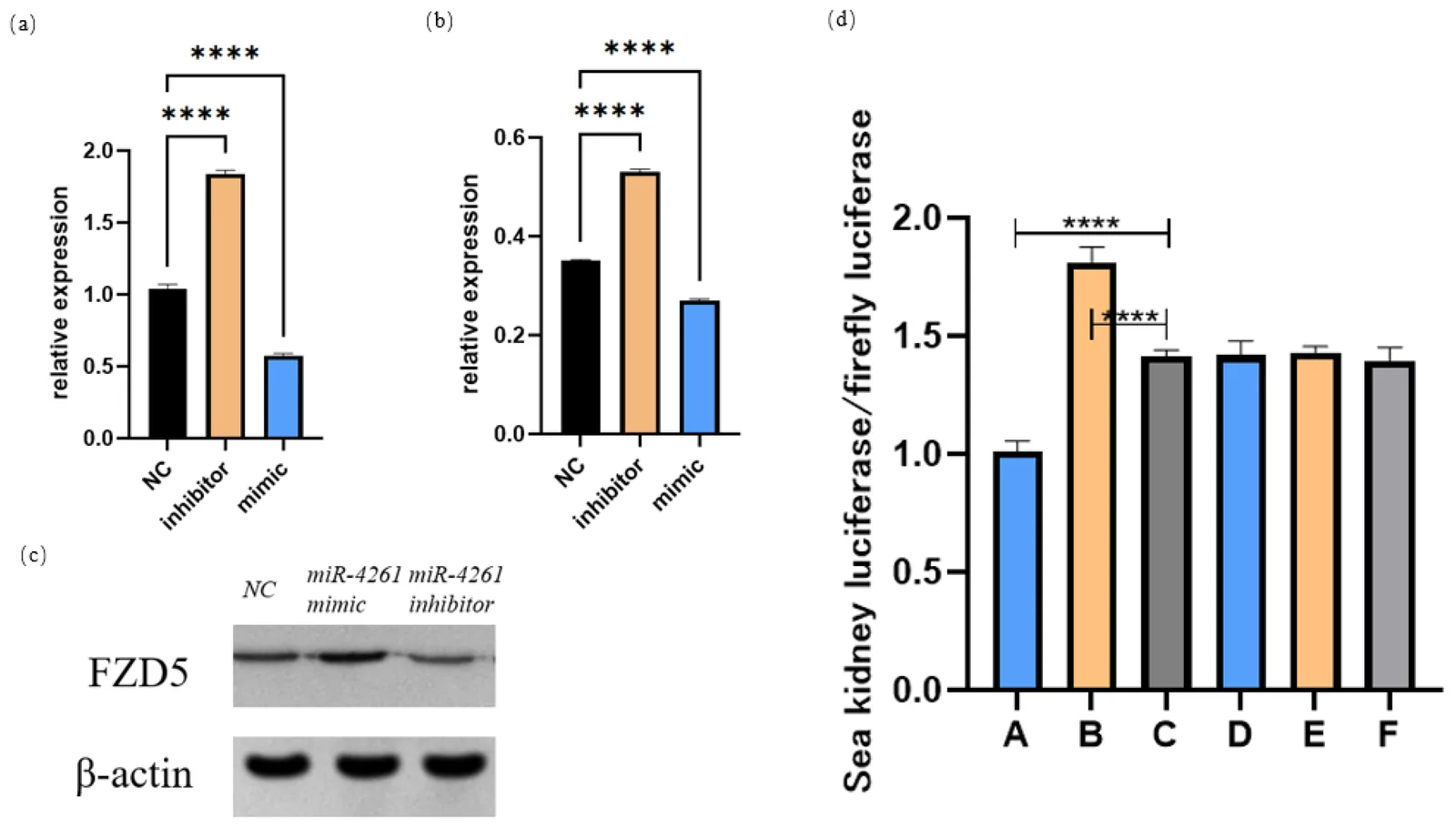
(**a**) qRT-PCR verification of FZD5 in KGN cells transfected with miR-4261 mimic/inhibitor/NC. The level of FZD5 mRNA was significantly lower than that of miR-NC (**** *p* < 0.0001), and the expression level of FZD5 mRNA in KGN cells transfected with miR-4261 inhibitor was significantly higher than that of miR-NC (**** *p* < 0.0001). (**b**) Western-Blot detection of FZD5 protein in KGN cells transfected with miR-4261 mimic, miR-4261 inhibitor and miR-NC. All Western blot experiments were performed in three independent biological replicates. Band gray values were quantified by ImageJ software, normalized to internal reference protein, and the relative expression fold change was statistically analyzed. The expression level of FZD5 protein in KGN cells transfected with −4261 mimic was significantly lower than that in miR-NC, while the protein expression level of FZD5 in KGN cells transfected with miR-4261 inhibitor was significantly higher than that in miR-NC (**** *p* < 0.0001). (**c**) Display of Western stripes. (**d**) Dual-luciferase reporter gene experiments in KGN cells. KGN cells co-transfected with wt-FZD5 and miR-4261 mimic/inhibitor exhibited fluorescence values. Attenuated/enhanced (**** *p* < 0.0001), KGN cells co-transfected with mut-FZD5 and miR-4261 mimic/inhibitor showed no significant difference in fluorescence value (*p* > 0.05). Groups A–F are listed in the table presented in Section 2.7 of the main text.

qRT-PCR was conducted to detect FZD5 mRNA expression in KGN cells transfected with the miR-4261 mimic, miR-4261 inhibitor or miR-NC. As shown in Figure 3a, FZD5 mRNA levels were significantly reduced in cells receiving the miR-4261 mimic compared with miR-NC (*p* < 0.0001). By contrast, FZD5 mRNA expression was markedly elevated in cells transfected with the miR-4261 inhibitor versus miR-NC (*p* < 0.0001). These data indicate that miR-4261 suppresses FZD5 expression at the mRNA level, implying FZD5 as a potential downstream target of miR-4261.

To confirm whether FZD5 acts as a downstream target of miR-4261 in KGN cells, western blot analysis was used to measure FZD5 protein abundance. Consistently, FZD5 protein expression was significantly lower in the miR-4261 mimic group and significantly higher in the miR-4261 inhibitor group relative to miR-NC. These findings demonstrate that miR-4261 modulates FZD5 expression at the protein level and further support FZD5 as a downstream target of miR-4261 (Figure 3b,c).

Dual-luciferase reporter assays were performed in KGN cells to validate the direct interaction between miR-4261 and FZD5. As illustrated in Figure 3d, co-transfection of wt-FZD5 with the miR-4261 mimic reduced luciferase activity, whereas co-transfection with miR-4261 inhibitor increased luciferase activity(**** *p* < 0.0001). No significant changes in luciferase activity were observed when mut-FZD5 was co-transfected with the miR-4261 mimic or inhibitor (*p* > 0.05). This result reveals that mutation within the FZD5 3′-UTR abolishes the regulatory effect of miR-4261 on reporter activity.

### 3.3. Effects of FZD5 on the Proliferation of Granulosa Cells

**Figure 4:**
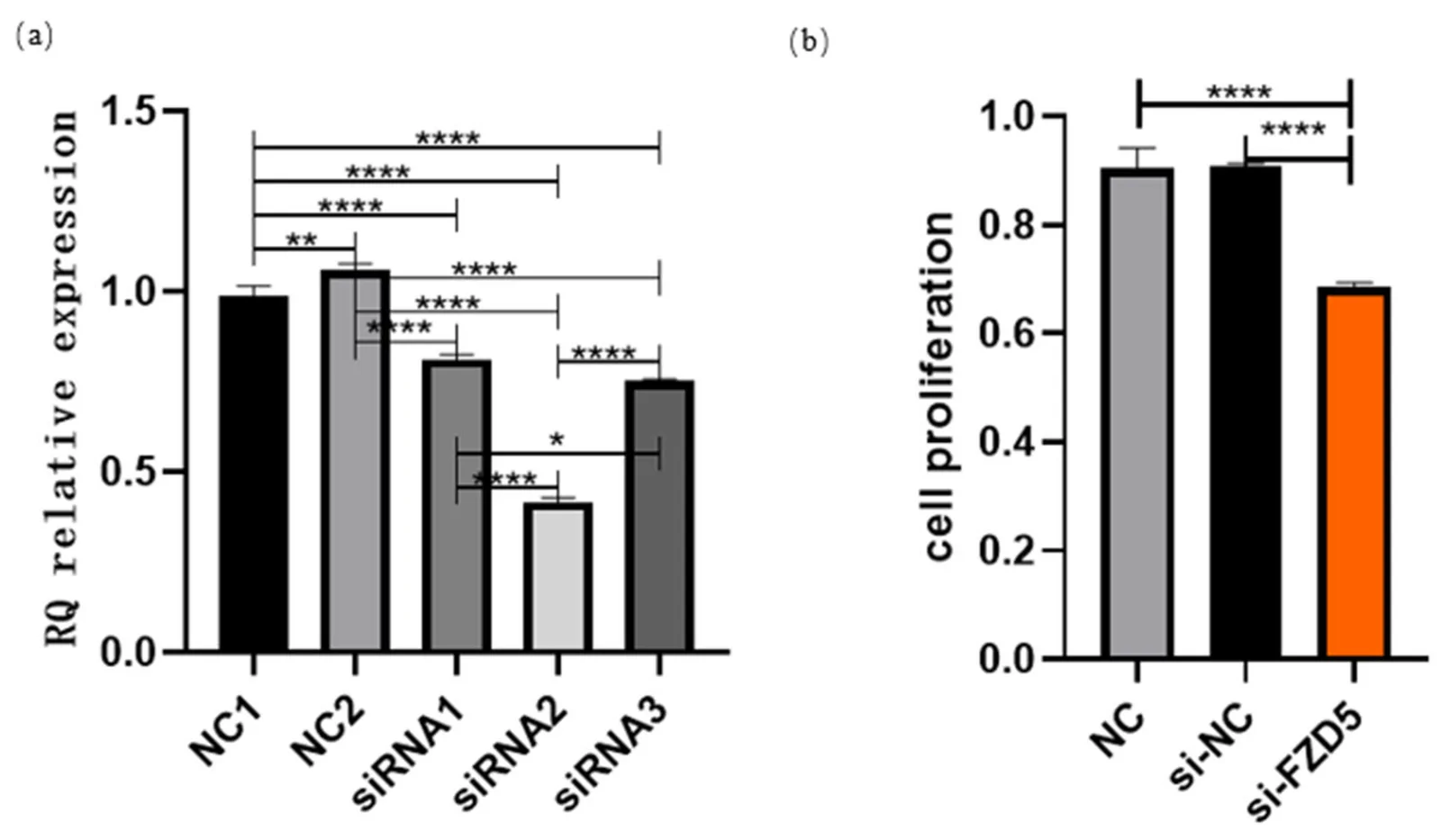
FZD5 knockdown impairs basal proliferation in KGN cells. (**a**) Quantitative RT–PCR analysis of *FZD5* mRNA expression in KGN cells 48 h after transfection with negative control siRNA (NC) or two independent *FZD5*-targeting siRNAs (si-FZD5-1 and si-FZD5-2). si-FZD5-2 achieved the most efficient knockdown (vs. NC **** *p* < 0.0001, ** *p* < 0.01, * *p* < 0.05) and was selected for subsequent functional assays. Data are presented as mean ± SD from three independent experiments. (**b**) Cell proliferation assay (CCK-8) performed 48 h post-transfection. Knockdown of *FZD5* significantly suppressed KGN cell proliferation compared with NC **** *p* < 0.0001), indicating that endogenous *FZD5* is required for maintaining baseline proliferative capacity.

First, specific small interfering RNAs (siRNAs) were used to knock down FZD5 expression in KGN cells, followed by validation of knockdown efficiency. Three FZD5-targeting siRNA sequences, designated siRNA1, siRNA2, and siRNA3, were synthesized. As shown in Figure 4a, 48 h after transfection, FZD5 expression in the siRNA2 group was significantly reduced compared with the negative control and blank groups (Figure 4a, *p* = 0.01). Accordingly, siRNA2 was selected as siFZD5 for all subsequent experiments.

As illustrated in Figure 4b, KGN cells transfected with si-FZD5 exhibited markedly suppressed proliferative capacity at 48 h (**** *p* < 0.0001). This finding demonstrates that FZD5 knockdown inhibits granulosa cell proliferation.

### 3.4. Effects of miR-4261 and FZD5 Co-Transfection on Proliferation of Granulosa Cells

Rescue experiments were performed to confirm whether miR-4261 regulates granulosa cell proliferation by targeting FZD5. KGN cells were co-transfected with miR-4261 inhibitor/mimic and wild-type or siRNA-mediated knockdown FZD5 constructs for functional verification.

**Figure 5:**
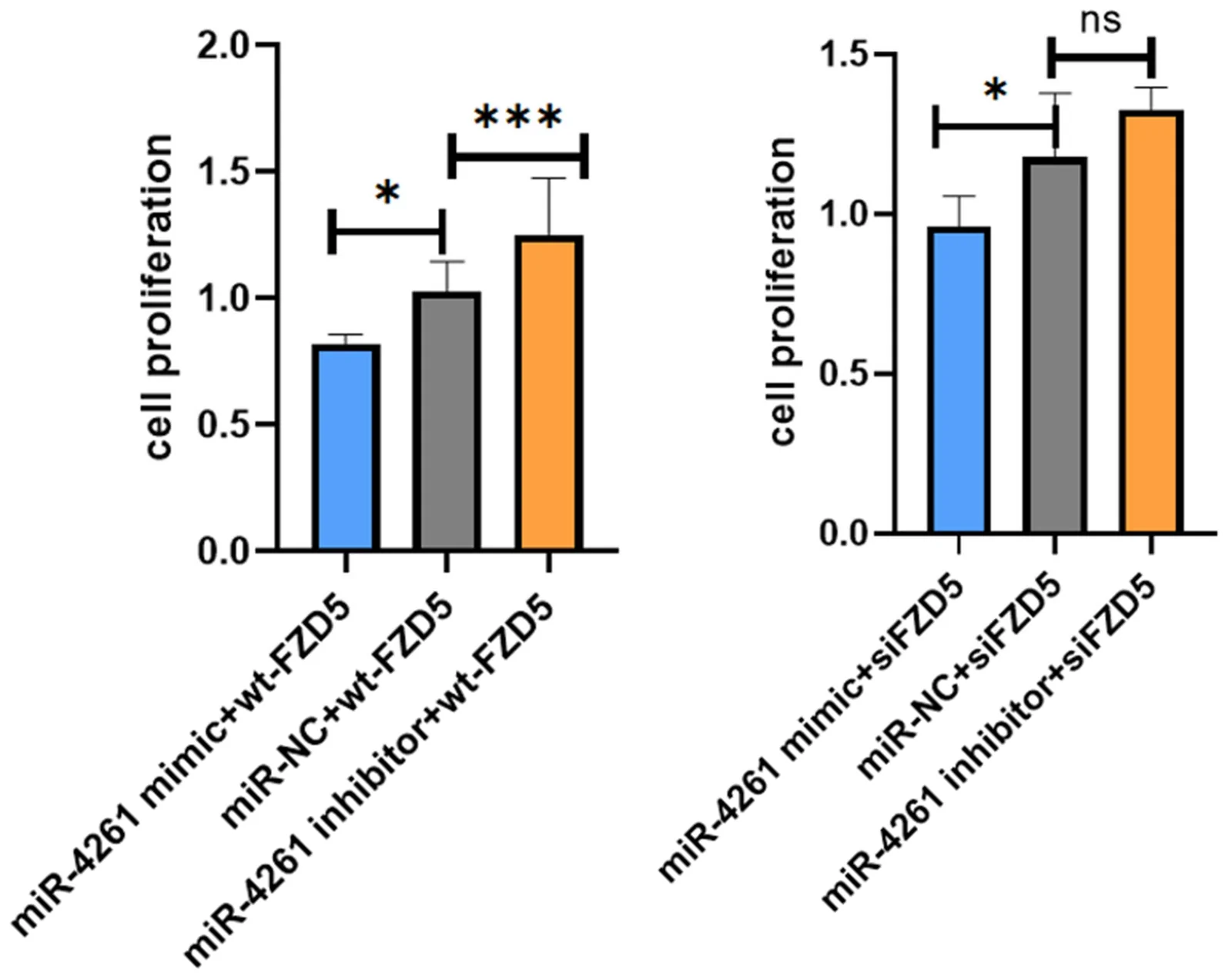
FZD5 is a functionally essential downstream effector of miR-4261 in regulating KGN cell proliferation. KGN cells were co-transfected with indicated combinations: (i) miR-NC or miR-4261 inhibitor + wild-type *FZD5* plasmid (wt-FZD5); or (ii) miR-NC or miR-4261 inhibitor + *siFZD5*. Proliferation was assessed at 48 h. In the presence of wt-FZD5, miR-4261 inhibition significantly enhanced proliferation vs. miR-NC (*** *p* < 0.001, * *p* < 0.05), suggesting that endogenous miR-4261 constrains proliferation via *FZD5* suppression. When *FZD5* was concurrently knocked down (*siFZD5*), the pro-proliferative effect of miR-4261 inhibition was completely abolished—no significant difference was observed between miR-4261 inhibitor and miR-NC groups (ns, *p* > 0.05). This epistasis result confirms that the functional output of miR-4261 modulation depends on *FZD5* expression, supporting *FZD5* as a necessary and rate-limiting mediator of miR-4261 activity in this cellular context. Data are presented as mean ± SD from three independent experiments. Statistical significance was determined by one-way ANOVA with Tukey’s post hoc test.

As shown in Figure 5, cells co-transfected with miR-4261 inhibitor and wt-FZD5 displayed significantly enhanced proliferation compared with cells co-transfected with miR-NC and wt-FZD5, further verifying the inhibitory effect of miR-4261 on granulosa cell growth. Notably, when endogenous FZD5 was depleted by siFZD5, the proliferative difference between the miR-4261 inhibitor group and the miR-NC group was completely abrogated. These results indicated that the pro-proliferative effect induced by miR-4261 inhibition was fully dependent on FZD5 expression, confirming that miR-4261 modulates granulosa cell proliferation primarily via targeting FZD5. Furthermore, miR-4261 mimic transfection still partially suppressed cell proliferation even after FZD5 knockdown, suggesting that FZD5 serves as a major, but not exclusive, functional target mediating the proliferative regulation of miR-4261 in granulosa cells.

## 4. Discussion

Our study identifies miR-4261 as a progesterone-regulated microRNA that inhibits human granulosa cell (GC) proliferation through direct repression of *FZD5*, a key receptor in the canonical Wnt/β-catenin pathway. This represents the first evidence linking progesterone signaling to Wnt/FZD axis modulation in ovarian follicular development.

In 2009, miR-4261 was first reported to be expressed in human embryonic stem cells and neural precursor cells [8]. Subsequent studies of miR-4261 have largely centered on its roles in targeted therapy for colorectal and esophageal cancers. At present, there is no relevant report on the study of miR-4261 on ovarian function, follicle development and granulosa cell function. Our study is the first to explore the effect of miR-4261 on the proliferative function of granulosa cells. Progesterone promotes the proliferation of granulosa cells by downregulating the expression of miR-4261, which is beneficial to the development and maturation of follicles. miR-4261 inhibits the proliferation of granulosa cells while promoting the proliferation of colorectal cancer and esophageal cancer cells, which suggests that miR-4261 has different proliferation regulation directions for different cell lines. Context-dependent miRNA function has been well-documented in previous literature. For instance, the let-7 miRNA family promotes proliferation of growth plate chondrocytes while suppressing proliferation in non-small-cell lung carcinoma cells [9,10]. Such divergent regulatory effects likely stem from distinct downstream target gene networks in different cellular contexts.

Gene Ontology (GO) and Kyoto Encyclopedia of Genes and Genomes (KEGG) enrichment analyses of predicted miR-4261 target genes identified FZD5 as a candidate targe. Through the detection of FZD5 mRNA and protein levels in KGN cells transfected with miR-4261 and the dual luciferase reporter gene assay, we confirmed that FZD5 is one of the direct downstream target genes of miR-4261. To further elucidate the molecular mechanism by which miR-4261 suppresses granulosa cell proliferation, we investigated the functional role of FZD5 in regulating KGN cell growth. siRNA-mediated FZD5 knockdown markedly reduced the proliferative capacity of KGN cells. Rescue co-transfection experiments showed that cells co-transfected with wt-FZD5 and miR-4261 inhibitor exhibited significantly enhanced proliferation compared with the wt-FZD5 + miR-NC group. However, after endogenous FZD5 was silenced by siFZD5, the proliferative difference between the miR-4261 inhibitor group and miR-NC group was completely abolished. These findings demonstrate that miR-4261 downregulation promotes granulosa cell proliferation by elevating FZD5 expression. Moreover, KGN cells co-transfected with the miR-4261 mimic and siFZD5 still showed significantly impaired proliferation compared with the miR-NC + siFZD5 group, indicating that FZD5 is not the sole target responsible for miR-4261-mediated proliferative regulation in granulosa cells.

FZD5 is one of the FZD receptors, belonging to the 7-transmembrane protein receptor family. The extracellular N-terminus contains a cysteine-rich domain, and its main signaling pathway is the Wnt/FZD signaling pathway [11]. Wnt proteins bind to membrane-localized FZD receptors via autocrine or paracrine modes, triggering intracellular signaling cascades that modulate target gene transcription and participate in diverse physiological and pathological processes. Upon Wnt ligand binding, FZD forms a complex with low-density lipoprotein receptor-related protein (LRP) 5/6, a single-pass transmembrane protein of the LRP family. The Wnt pathway is categorized into the canonical β-catenin-dependent pathway and non-canonical β-catenin-independent pathway. To date, ten FZD receptors have been identified in humans. The Wnt signaling pathway participates in the regulation of a wide spectrum of biological processes [12]. Wnt5a, for instance, interacts with FZD5 to mediate multiple inflammatory disorders, including rheumatoid arthritis, psoriasis, and sepsis [13,14,15]. These reports support Wnt5a as a pro-inflammatory marker, while FZD5 serves as the indispensable membrane receptor required for Wnt ligand function. Paradoxically, Kelly demonstrated that Wnt5a suppresses inflammatory responses by downregulating tumor necrosis factor-α (TNF-α) receptor expression in intestinal epithelial cells [16], suggesting that the Wnt/FZD pathway exerts context-dependent effects across distinct inflammatory diseases. Beyond inflammatory conditions, the Wnt/FZD5 pathway also exhibits divergent functions in human malignancies. Taye et al. reported aberrant activation of the Wnt/FZD5 pathway during colorectal cancer invasion and metastasis, and FZD5 protein expression was strongly correlated with the clinicopathological features of colorectal cancer [17,18]. However, Dong found that FZD5 could prevent epithelial-mesenchymal transition (EMT) in gastric cancer, leading to better prognosis [19]. Many scholars have found that FZD5 acts as both a tumor promoter and a tumor suppressor [20,21]. This dual regulatory property of FZD5 helps explain the opposite effects of miR-4261 in tumor cells versus granulosa cells, and this unique characteristic of FZD5 is one primary rationale for selecting this target in our present study.

At present, there are no reports on the application of FZD5 in the reproductive field and few reports on the functional regulation of the Wnt/FZD signaling pathway and human granulosa cells. The main reports focus on the abnormal expression of Wnt signaling, which was related to inflammation in PMOS [22,23,24]. How Wnt/FZD interact and regulate follicular microenvironment remains unclear. This study indicated that miR-4261 can further regulate the expression of FZD5, which indicates that progesterone may participate in the regulation of related signals on the Wnt/FZD5 signaling pathway. Our study identifies FZD5 as a functionally dominant, non-redundant target of miR-4261 for regulating progesterone secretion in human GCs. Although FZD5 is neither the sole enzyme nor the only signaling molecule involved in progesterone biosynthesis—STAR, CYP11A1, HSD3B2, and other Wnt receptors all play essential roles—specific repression of FZD5 by miR-4261 creates a bottleneck in Wnt/β-catenin-dependent transcriptional activation of steroidogenic genes. Thus, FZD5 should not be regarded as an isolated “master regulator” but rather a context-dependent critical node whose modulation exerts an outsized influence on the functional output of this pathway.

By suppressing miR-4261 expression, progesterone facilitates granulosa cell proliferation and supports follicular growth. Our in vitro experiments verified that miR-4261 directly targets FZD5 within the Wnt signaling cascade to modulate granulosa cell proliferation, identifying FZD5 as a potential downstream effector of progesterone in regulating granulosa cell function and follicle development. Future studies will further explore the downstream molecular mechanisms of miR-4261 and systematically clarify the regulatory roles of the Wnt/FZD5 pathway in granulosa cell proliferation, apoptosis, and steroid hormone secretion.

A key limitation of our study is the lack of in vivo validation. Our mechanistic data were generated in the KGN cell line—a model with well-documented utility in studying steroidogenesis and proliferation. While technically demanding, future work could employ a human granulosa cell xenograft model in immunodeficient mice, combined with timed gonadotropin stimulation and targeted miR-4261 modulation, to assess effects on graft growth, progesterone output, and follicle-like structure formation. Alternatively, conditional *FZD5* deletion in mouse granulosa cells can directly test the requirement of this pathway for endogenous folliculogenesis.

## 5. Conclusions

**Figure 6:**
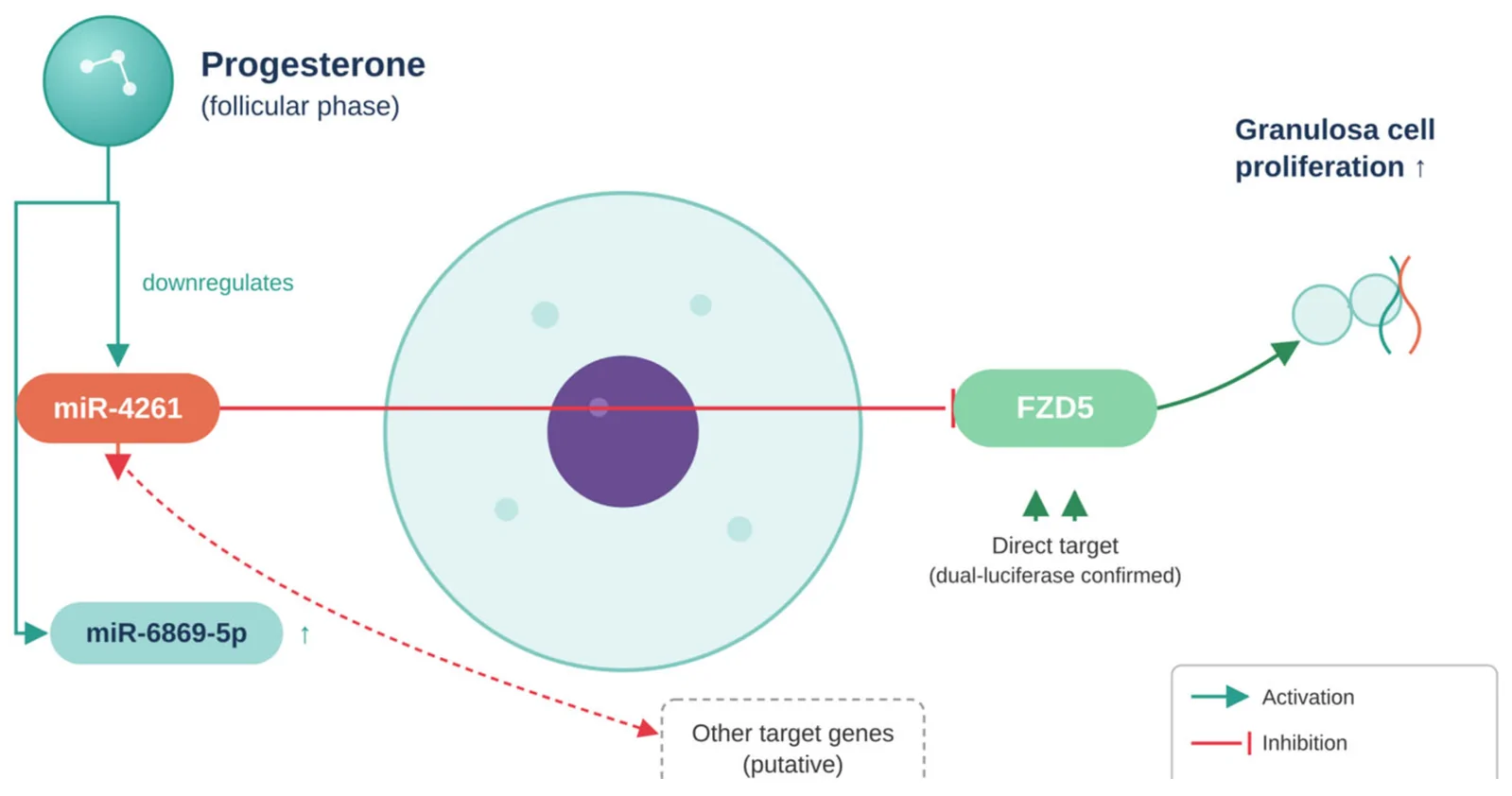
A schematic diagram summarizing the miR-4261/FZD5/progesterone regulatory pathway.

Our findings found that down-regulation of miR-4261 can promote the proliferation of granulosa cells. The knockdown of FZD5 can inhibit the proliferation of granulosa cells. FZD5 is one of the direct downstream target gene of miR-4261 in KGN cells. miR-4261 can regulate granulosa cell proliferation by targeting FZD5(Figure 6).

## Data Availability

All data and models generated or used during the study appear in the submitted article. The detailed datasets used or analyzed during the current study are available from the corresponding author on reasonable request.
